# Novel Evolution of Mineralocorticoid Receptor in Humans Compared to Chimpanzees, Gorillas, and Orangutans

**DOI:** 10.3390/genes15060767

**Published:** 2024-06-12

**Authors:** Yoshinao Katsu, Jiawen Zhang, Michael E. Baker

**Affiliations:** 1 Faculty of Science, Hokkaido University, Sapporo 060-0810, Japan; 2 Graduate School of Life Science, Hokkaido University, Sapporo 060-0810, Japan; jiawenzhang0829@foxmail.com; 3 Division of Nephrology-Hypertension, Department of Medicine, 0693, University of California, 9500 Gilman Drive, San Diego, La Jolla, CA 92093, USA; 4 Center for Academic Research and Training in Anthropogeny (CARTA), University of California, San Diego, La Jolla, CA 92093, USA

**Keywords:** mineralocorticoid receptor, DNA-binding domain, evolution, human MR, chimpanzee MR, aldosterone

## Abstract

We identified five distinct full-length human mineralocorticoid receptor (MR) genes containing either 984 amino acids (MR-984) or 988 amino acids (MR-988), which can be distinguished by the presence or absence of Lys, Cys, Ser, and Trp (KCSW) in their DNA-binding domain (DBD) and mutations at codons 180 and 241 in their amino-terminal domain (NTD). Two human MR-KCSW genes contain either (Val-180, Val-241) or (Ile-180, Val-241) in their NTD, and three human MR-984 genes contain either (Ile-180, Ala-241), (Val-180, Val-241), or (Ile-180, Val-241). Human MR-KCSW with (Ile-180, Ala-241) has not been cloned. In contrast, chimpanzees contain four MRs: two MR-988s with KCSW in their DBD, or two MR-984s without KCSW in their DBD. Chimpanzee MRs only contain (Ile180, Val-241) in their NTD. A chimpanzee MR with either (Val-180, Val-241) or (Ile-180, Ala-241) in the NTD has not been cloned. Gorillas and orangutans each contain one MR-988 with KCSW in the DBD and one MR-984 without KCSW, and these MRs only contain (Ile-180, Val-241) in their NTD. A gorilla MR or orangutan MR with either (Val-180, Val-241) or (Ile-180, Ala-241) in the NTD has not been cloned. Together, these data suggest that human MRs with (Val-180, Val-241) or (Ile-180, Ala-241) in the NTD evolved after humans and chimpanzees diverged from their common ancestor. Considering the multiple functions in human development of the MR in kidney, brain, heart, skin, and lungs, as well as MR activity in interaction with the glucocorticoid receptor, we suggest that the evolution of human MRs that are absent in chimpanzees may have been important in the evolution of humans from chimpanzees. Investigation of the physiological responses to corticosteroids mediated by the MR in humans, chimpanzees, gorillas, and orangutans may provide insights into the evolution of humans and their closest relatives.

## 1. Introduction

The mineralocorticoid receptor (MR) is a ligand-activated transcription factor, belonging to the nuclear receptor family, a diverse group of transcription factors that arose in multicellular animals [1,2,3,4,5]. The traditional physiological function of the MR is to maintain electrolyte balance by regulating sodium and potassium transport in epithelial cells in the kidney and colon [6,7,8,9,10]. In addition, the MR has important physiological functions in many other tissues, including the brain, heart, skin, and lungs [10,11,12,13,14,15,16,17].

**Figure 1:**
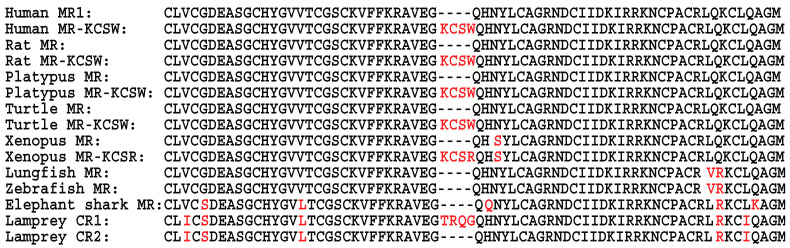
A comparison of the DNA-binding domain on human MR1, human MR-KCSW, rat MR, rat MR-KCSW, platypus MR, platypus MR-KCSW, turtle MR, turtle MR-KCSW, *Xenopus* MR, *Xenopus* MR-KCSR, lungfish MR, zebrafish MR, elephant shark MR, and lamprey CR1 and CR2.

The MR and its paralog, the glucocorticoid receptor (GR), descended from an ancestral corticoid receptor (CR) in a cyclostome (jawless fish) that evolved about 550 million years ago at the base of the vertebrate line [18,19,20,21,22,23,24,25]. A descendent of this ancestral steroid receptor, the CR in lamprey (*Petromyzon marinus*), is activated by aldosterone [26,27] and other corticosteroids [26,27]. Lampreys contain two CR isoforms, which differ only in the presence of a four amino acid insert Thr, Arg, Gln, and Gly (TRQG) in their DNA-binding domain (DBD) [28] (Figure 1). We found that several corticosteroids had a similar half-maximal response (EC50) for lamprey CR1 and CR2 [27]. However, these corticosteroids had a lower fold-activation of transcription for CR1, which contains the four amino acid insert, than for CR2 suggesting that the deletion of the four amino acid sequence in CR2 selected for increased transcriptional activation by corticosteroids of CR2 [27,28].

A distinct MR and GR first appear in sharks and other cartilaginous fishes (Chondrichthyes) [20,22,29,30,31,32]. The DBD in elephant shark MR and GR lacks the four amino acid sequence found in lamprey CR1 [27] (Figure 1). We inserted this four-residue sequence from lamprey CR1 into the DBD in elephant shark MR and GR and found that in HEK293 cells cotransfected with the TAT3 promoter, the mutant elephant shark MR and GR had lower transcriptional activation by corticosteroids than did their wild-type elephant shark MR and GR counterparts, indicating that the insertion of the four amino acid sequence into the DBD of wild-type elephant shark MR and GR had a similar effect on transcriptional activation as the KCSW insert had in the DBD of lamprey CR1 [28].

Based on these results with lamprey CR1 and CR2, we analyzed the DBD sequence of human MR, which had been cloned, sequenced, and characterized by Arriza et al. [33], and we found that like elephant shark MR, this human MR (MR1) lacks a four-residue segment in its DBD. This human MR has been widely studied [8,10,12,13,34,35,36]. Unexpectedly, our BLAST [37] search with the DBD from this human MR found a second, previously described, human MR with a KCSW insert (MR-KCSW) in its DBD [38,39,40] (Figure 1). A homology model of human MR with KCSW in the DBD constructed by Wickert et al. [40] found no distortion by the four amino acids on adjacent secondary structures of the DBD in human MR, consistent with activation of this MR by corticosteroids.

**Figure 2:**
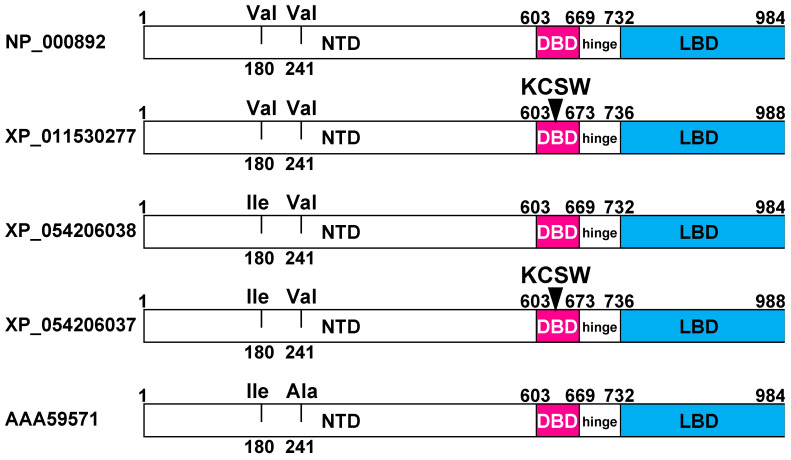
A comparison of five full-length human mineralocorticoid receptors. There are three human MRs with 984 amino acids. They contain either valine-180 and valine-241, isoleucine-180 and valine-241, or isoleucine-180 and alanine-241 in the amino-terminal domain (NTD). There are two human MRs with 988 amino acids and KCSW in the DBD and either valine-180 and valine-241, or isoleucine-180 and valine-241 in the NTD. A human MR with KCSW and isoleucine-180 and alanine-241 in the NTD was not found in GenBank.

As described later, further BLAST searches found two full-length human MRs with this insert (MR-KCSW) and three full-length human MRs without this insert. The three human MRs without the KCSW insert contain either (Ile-180, Ala-241), (Val-180, Val-241), or (Ile-180, Val-241) in their amino terminal domain (NTD) (Figure 2). The two human MR-KCSW splice variants contain either (Val-180, Val-241) or (Ile-180, Val-241) in their NTD (Figure 2). A human MR-KCSW with (Ile-180, Ala-241) has not been cloned.

Here, in this brief report, we describe our evolutionary analysis of human MR from a comparison of five full-length human MRs with four full-length MRs in chimpanzees, and two full-length MRs in gorillas and orangutans. We find that chimpanzees, gorillas, and orangutans lack an MR with either (Ile-180, Ala-241) or (Val-180, Val-241) in their NTD. We propose that MRs with these amino acids in the NTD evolved in humans after the divergence of humans from chimpanzees. Due to the multiple functions in human development of the MR alone [11,15,16,41], as well as due to the interaction of the MR with the GR [17,42,43,44,45,46], we suggest that the evolution of three distinct MRs in humans that are absent in chimpanzees may have been important in the evolution of humans from chimpanzees.

The DBD of the human MR-KCSW splice variant has an insertion of four amino acids that is absent in human MR1. Otherwise, the rest of the sequences of human MR and human MR-KCSW are identical. Differences between the DBD sequence in human MR and selected vertebrate MRs are shown in red. Protein accession numbers are AAA59571 for human MR; XP_011530277 for human MR-KCSW; NP_037263 for rat MR; XP_038953451 for rat MR-KCSW; XP_007669969 for platypus MR; XP_016083764 for platypus MR-KCSW; XP_043401725 for turtle MR; XP_037753298 for turtle MR-KCSW; NP_001084074 for *Xenopus* MR; XP_018098693 for *Xenopus* MR-KCSR; BCV19931 for lungfish MR; NP_001093873 for zebrafish MR; XP_007902220 for elephant shark MR; XP_032811370 for lamprey CR1; and XP_032811371 for lamprey CR2.

## 2. Methods

The basic alignment search tool (BLAST) [37,47] was used to search GenBank with the sequence of human MR (Accession AAA59571) for similar proteins in humans, chimpanzees, gorillas, and orangutans. We also retrieved MR sequences from rat and platypus, as well as two basal vertebrates: *Xenopus laevis* and turtles. We also retrieved MR sequences from ancestors of terrestrial vertebrates: lungfish, elephant shark, and lamprey. The DBDs of these vertebrates are shown in Figure 1.

## 3. Results and Discussion

### 3.1. Novel Mineralocorticoid Receptors in Humans

A BLAST [37,47] search of GenBank retrieved five distinct full-length human MR genes (Figure 2), which are identical in the LBD and hinge segment, but differ in the DBD and NTD. Two full-length human MRs, with 988 amino acids, contain a KCSW insert in the DBD (Figure 2), which is absent in the human MR with 984 amino acids cloned by Arriza et al. [33] (Figure 2), as well as two other full-length MRs with 984 amino acids (Figure 2). These three human MRs with 984 amino acids contain either (Ile-180, Ala-241), (Val-180, Val-241), or (Ile-180, Val-241) in the NTD (Figure 2). The two human MR-KCSWs contain either (Val-180, Val-241) or (Ile-180, Val-241) in their NTD. A human MR-KCSW sequence with (Ile-180, Ala-241) has not been deposited in GenBank.

### 3.2. Chimpanzees Have Four Full-Length Mineralocorticoid Receptors

**Figure 3:**
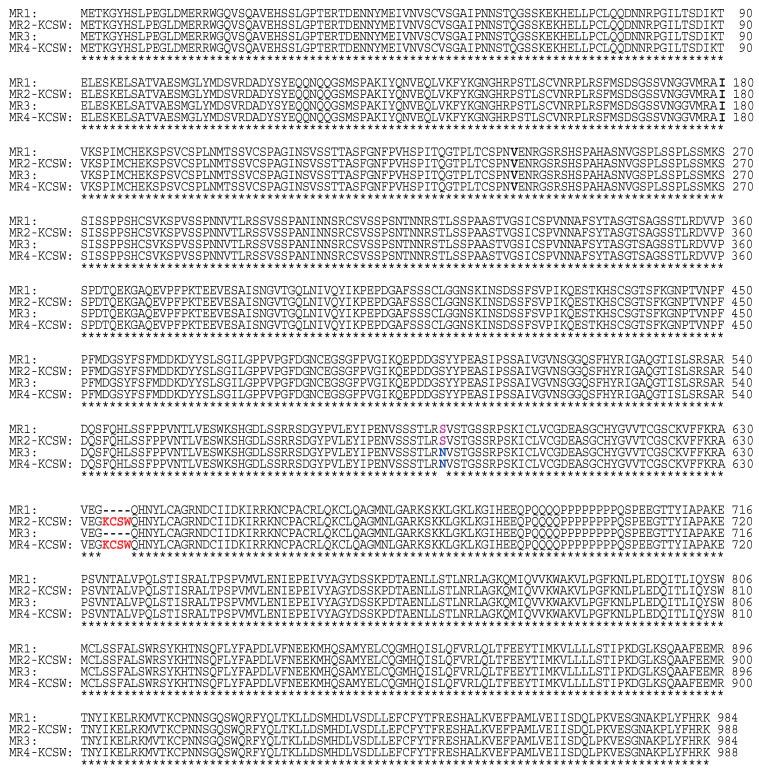
A multiple sequence alignment of four chimpanzee mineralocorticoid receptors. The four chimpanzee MRs were aligned with Clustal Omega [48]. In contrast to human MRs, chimpanzee MRs only contain an isoleucine at codon 180 and a valine at codon 241. Chimpanzees also contain an MR with either a serine or an asparagine at codon 591, while human MRs only contain a serine at codon 591.

Our BLAST search retrieved four full-length chimpanzee MRs (Figure 3). Two of these chimpanzee MRs have DBDs that are identical to the DBD in human MRs without the KCSW insert (Figure 1 and Figure 3), and two chimpanzee MRs have DBDs that are identical to the DBD in human MR-KCSW (Figure 1 and Figure 3). All four chimpanzee MRs contain (Ile-180, Val-241) in their NTD, which is similar to the NTD in one human MR (Figure 2). Chimpanzees lack an MR with either (Ile-180, Ala-241) or (Val-180, Val-241) in their NTD. The different chimpanzee MR sequences contain either a Ser-591 or an Asn-591 in their NTD (Figure 3). Human MRs contain a Ser-591 corresponding to Ser-591 in chimpanzee MR. Our BLAST search did not find a human MR with Asn-591 in the NTD.

### 3.3. Gorillas and Orangutans Have Two Full-Length MRs

**Figure 4:**
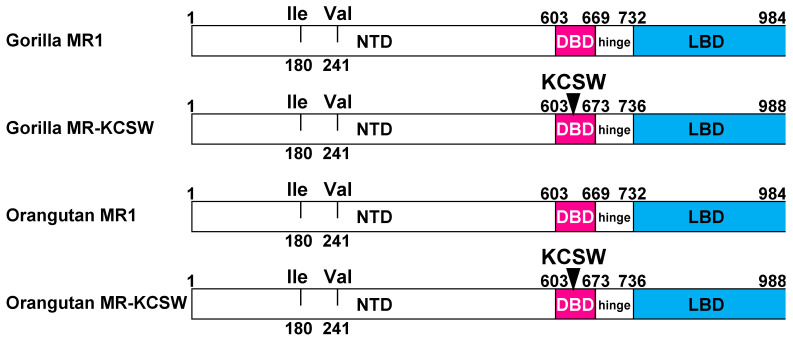
A comparison of the gorilla and orangutan MRs. The NTD of gorilla MRs and orangutan MRs conserve an isoleucine-180 and valine-241 pair corresponding to isoleucine-180 and valine-241 in the NTD of human MR. A gorilla MR or an orangutan MR with either valine-180 and valine-241 or isoleucine-180 and alanine-241 in the NTD was not found in GenBank. Gorillas and orangutans also only contain an MR with a serine at codon 591, unlike chimpanzee MRs, which contain either serine at codon 591 or asparagine at codon 591. Human MRs only contain a serine at codon 591.

Gorillas and orangutans each contain two full-length MRs: one MR contains 984 amino acids and one MR contains 988 amino acids (Figure 4). The MRs with 988 amino acids have the KCSW sequence in the DBD. All full-length gorilla MRs and full-length orangutan MRs have isoleucine-180 and valine-241 in the amino-terminal domain (NTD) (Figure 4). A gorilla MR or orangutan MR with either valine-180 and valine-241 or isoleucine-180 and alanine-241 in the NTD was not found in GenBank.

### 3.4. Evolutionary Divergence of Human and Chimpanzee Mineralocorticoid Receptors

Our analysis of human and chimpanzee MRs indicates that a human MR with either (Ile-180, Ala-241) or (Val-180, Val-241) in the NTD evolved after the divergence of humans and chimpanzees from a common ancestor. The physiological consequences of (Ile-180, Ala-241) or (Val-180, Val-241) in the NTD of human MR remain to be elucidated. Moreover, the absence of an asparagine at codon 591 in human MR is intriguing considering the presence of an asparagine at codon 591 in the MR in chimpanzees. As a first step, to determine if there are differences in transcriptional activation of these MRs by corticosteroids, we are cloning these human and chimpanzee MR genes and are screening them for transcriptional activation by aldosterone, cortisol, and other corticosteroids. We will also screen MR-GR dimers for activation by aldosterone and other corticosteroids [28,42,43,44,45,49], which is an important mechanism for regulating corticosteroid activity in the brain [15,17,44,50,51].

### 3.5. Evolution of the Mineralocorticoid Receptor DBD in Basal Terrestrial Vertebrates

The evolution of an MR with a DBD containing a four amino acid insert in human MR and other terrestrial MRs was surprising to us (Figure 1). We did not expect to find the KCSW insert in human MR at the position homologous to the position of TRQG in the DBD of lamprey CR because an insert at this position was not present in the DBD of elephant sharks and lungfish MRs (Figure 1). Moreover, the rest of the DBD sequence is highly conserved in terrestrial vertebrates (Figure 1), suggesting that the evolution of a four amino acid sequence in the DBD has an important function in terrestrial vertebrates. The evolution of the KCSR sequence into the DBD *Xenopus* MR (Figure 1) places the re-emergence of this motif in the MR close to the origin of terrestrial vertebrates, in which evolution of aldosterone synthesis in lungfish had an important role in the conquest of land by terrestrial vertebrates [4,6,52,53,54,55,56,57,58]. The evolution of KCSR in *Xenopus* MR in the position homologous to the DBD in human MR suggests that insertion of KCSR in *Xenopus* MR also may have been important early in the conquest of land by terrestrial vertebrates. Moreover, turtles contain an MR with KCSW in the position in DBD (Figure 1) homologous to human MR DBD, indicating that KCSW also has an ancient origin in MRs in terrestrial vertebrates. Indeed, the conservation of KCSW in the MR DBD in turtles and humans suggests an important function for KCSW in terrestrial vertebrates. 

## Data Availability

The data presented in this study are available upon request from the corresponding author.
